# Neglected malaria parasites in hard-to-reach areas of Odisha, India: implications in elimination programme

**DOI:** 10.1186/s12936-021-04010-8

**Published:** 2021-12-23

**Authors:** Madhusmita Bal, Ramakanta Rana, Arundhuti Das, Hemant Kumar Khuntia, Nilam Somalkar, Niranjan Sahoo, Jyoti Ghosal, Sanghamitra Pati, Ambarish Dutta, Manoranjan Ranjit

**Affiliations:** 1 ICMR-Regional Medical Research Center, Chandrasekharpur, Bhubaneswar, 751023 Odisha India; 2 Regional Office for Health & Family Welfare, Govt. of India, Bhubaneswar, Odisha India; 3 Odisha University of Agriculture and Technology, Bhubaneswar, Odisha India; 4 Indian Institute of Public Health, Bhubaneswar, Odisha India

**Keywords:** *Plasmodium ovale* spp., *Plasmodium malariae*, PCR, Hard-to reach areas, Malaria elimination, Odisha, India

## Abstract

**Background:**

Information on the foci of *Plasmodium* species infections is essential for any country heading towards elimination. Odisha, one of the malaria-endemic states of India is targeting elimination of malaria by 2030. To support decision-making regarding targeted intervention, the distribution of *Plasmodium* species infections was investigated in hard-to-reach areas where a special malaria elimination drive, namely *Durgama Anchalare Malaria Nirakaran* (DAMaN) began in 2017.

**Methods:**

A cross-sectional survey was conducted in 2228 households during July to November 2019 in six districts, to evaluate the occurrence of *Plasmodium* species. The species were identified by polymerase chain reaction (PCR) followed by sequencing, in case of *Plasmodium ovale*.

**Results:**

Of the 3557 blood specimens tested, malaria infection was detected in 282 (7.8%) specimens by PCR. Of the total positive samples, 14.1% were *P.* *ovale* spp. and 10.3% were *Plasmodium malariae* infections. The majority of *P.* *ovale* spp. (75.8%) infections were mixed with either *Plasmodium falciparum* and/or *Plasmodium vivax* and found to be distributed in three geophysical regions (Northern-plateau, Central Tableland and Eastern Ghat) of the State, while *P. malariae* has been found in Northern-plateau and Eastern Ghat regions. Speciation revealed occurrence of both *Plasmodium* *ovale curtisi* (classic type) and *Plasmodium ovale wallikeri* (variant type).

**Conclusions:**

In the present study a considerable number of *P. ovale* spp. and *P. malariae* were detected in a wide geographical areas of Odisha State, which contributes around 40% of the country’s total malaria burden. For successful elimination of malaria within the framework of national programme, *P. ovale* spp. along with *P. malariae* needs to be incorporated in surveillance system, especially when *P. falciparum* and *P. vivax* spp. are in rapid decline.

## Background

Malaria is one of the most important global public health challenges that contributes to a high burden of mortality and morbidity [1, 2]. According to the World Malaria Report [1, 2] an estimated 215 million malaria cases and 384,000 malaria deaths were reported globally in 2019. Amongst them, 3% are from Southeast Asia and the majority are from India [3]. Of the five *Plasmodium* species causing malaria in humans *Plasmodium falciparum* is most virulent and responsible for most fatalities [4]. *Plasmodium vivax,* once known to cause relapse, has recently been found to be associated with severe clinical presentations and deaths as well [5]. *Plasmodium malariae*, an established cause of nephrotic syndrome, can lead to progressive renal failure, particularly in adolescents or young adults [6]. *Plasmodium knowlesi*, a simian parasite, has recently been detected in humans, causing severe disease and fatalities [7]. *Plasmodium ovale* causes clinically mild disease but possesses the ability to cause relapse, like *P. vivax* [8]. Molecular fingerprinting has revealed that ovale malaria is caused by two genetically distinct sub-species *Plasmodium* *ovale wallikeri* and *Plasmodium* *ovale curtisi* [9]. Historically, *P. vivax* was the predominant species causing malaria in India (> 53%), but in the recent past the proportion *P. falciparum* has increased to 64%, while *P. malariae* remained around 2% [10, 11]. *Plasmodium ovale* spp. has been formally reported on only two occasions from India [12, 13]. *Plasmodium knowlesi* has not been reported from India so far, but *P. knowlesi-*specific gene sequences have been reported in the malaria vector *Anopheles sundaicus* in Andaman and Nicobar Islands [14].

With 4% of the total population of India, the state of Odisha, with its humid and hot climatic conditions and forested low-hill topography, is highly conducive to malaria transmission, and contributes to around 40% of the country’s total burden of malaria, 91.5% of which is caused by *P. falciparum* and 8.5% by non-falciparum species [15]. The National Framework for Malaria Elimination (NFME) launched by Government of India in 2016 set the goal to eliminate malaria from the entire country by 2030 [16]. Realising this goal as a significant public health challenge, Odisha State chapter of National Vector-borne Disease Control Programme (NVBDCP), which is responsible for malaria control activities in the state, in addition to national initiatives, launched in 2017 a special drive named *Durgama Anchalare Malaria Nirakaran* (translation: malaria elimination in less accessible areas), in short DAMaN.

DAMaN targets high-transmission, recalcitrant malaria ‘pockets’, which were identified in hard-to-reach areas of the state, spread over 23 districts. DAMaN comprised half-yearly mass malaria screening of populations living in villages and hamlets in hard-to-reach areas with historically high malaria burden, less access to routine malaria programme, and complete treatment of malaria positive cases. It also comprised distribution of long-lasting insecticidal nets (LLIN) to large sections of the state population and actively promoted their use. The details of DAMaN are described elsewhere [17]. Although the state has witnessed a drastic reduction in falciparum malaria cases (> 80%) after launching of DAMaN, malaria continues to persist in many hard-to-reach areas [18]. This has led the state to initiate a neutral assessment of DAMaN, which has been described elsewhere [17]. Effective malaria elimination strategy needs not only continuous surveillance of current levels of transmission but also monitoring of the hidden reservoir of other less common malaria species that are often associated with persistent transmission of the disease. Rapid diagnostic tests (RDTs) used in malaria elimination programmes are easy and reliable tools for diagnosis of either *P. falciparum* or *P. vivax*, but other species are often unrecognized or underestimated, due to low parasite densities, technical difficulties and observational error [19]. Routine diagnostic tests may not detect rarer malaria-causing species, which may lead to their unchecked transmission.

Recently a study conducted in Tanzania has revealed persistent transmission of *P. malariae* and *P. ovale* spp. in areas of declining *P. falciparum* transmission [6]. Whether this scenario is also likely to emerge in traditionally high malaria transmission areas of India that are witnessing a rapid decline in falciparum malaria remains to be seen. Emergence of such situations can have far-reaching public health ramifications, especially for national malaria elimination initiatives, because the transmission of rarer species, also referred as ‘neglected malaria parasites’, is likely to remain undetected and unaddressed by current routine programme measures. This can lead to their persistence in the community, which can continue to fuel transmission of malaria in the country, jeopardizing ambitious elimination goals. Against the backdrop of this apprehension, the decline of falciparum malaria in DAMaN-implemented areas of Odisha afforded the current study the opportunity to examine the presence of less common/neglected malaria species, so that the programme can be informed about their presence, if any. If found present, this can lead to the mounting of control/elimination measures targeted against such neglected malaria species in addition to the routine focus on falciparum and vivax malaria*.* As a part of the DAMaN assessment study, this current study aimed to identify the presence of different types of malaria species in the communities covered by DAMaN.

## Methods

### Study setting

**Fig. 1:**
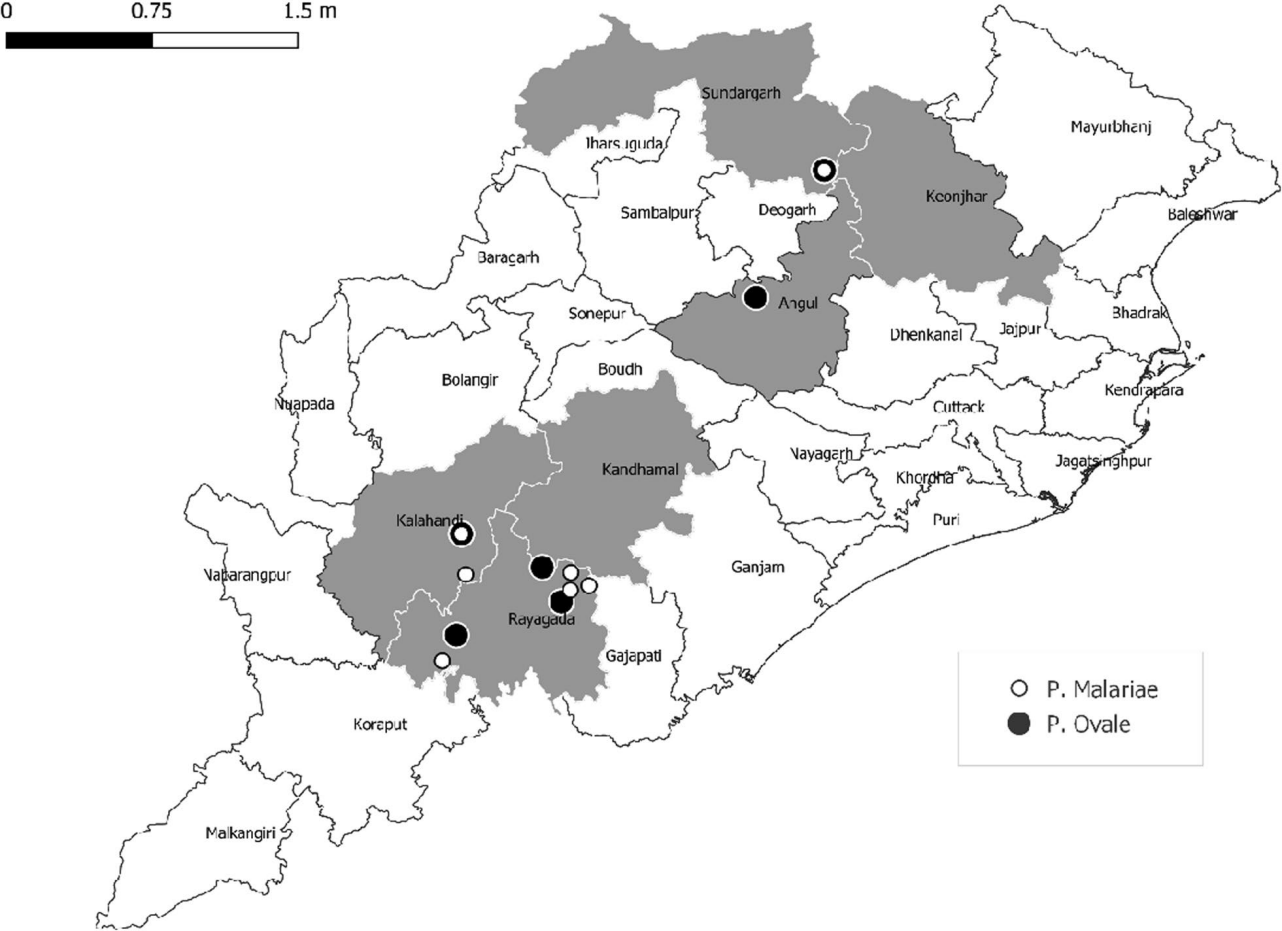
Map of Odisha showing sample collection site from six DAMaN implemented Districts (grey). Each site is represented by two blocks comprising 16 sub-centres. Both mono and mixed species infection due to *Plasmodium* were observed in all five districts (out of six). But *P. ovale* spp. and *P. malariae* was found in four study districts. *In Sundargarh and Kalahandi district there was an overlap of symbols as both *P. ovale* spp. and *P. malariae* were detected at the same GIS coordinate

DAMaN is being implemented in 23 districts out of 30 administrative districts of the State of Odisha [17]. The current study took place in six DAMaN-implementing districts representing three distinct geophysical regions (Northern plateau: Sundargarh and Keonjhar district, Central table land: Anugul and Kandhamal district and Eastern ghat: Raygada and Kalahandi district) (Fig. 1). The districts have been selected based on the annual parasite index (API), the number of confirmed cases of malaria per 1,000 population reported by NVBDCP-Odisha in 2015.

A survey was conducted at 2,228 households in those six districts between August 2019 and February 2020. One out of six sampled households had pregnant women and one out of two had children under 5 years of age. The average distance of these villages from ICMR-RMRC, Bhubaneswar laboratory is around 300 km. Blood samples were obtained from 3557 consenting individuals who were present at the time of the survey in the selected households.

### Blood specimen collection and processing

During this survey 1 ml of venous blood sample was collected in BD Vacutainer ® EDTA tubes. The blood samples collected from the field sites were preserved at 4 ℃ at the local hospital and transported to the ICMR-RMRC laboratory within 24 h. The Pf-PAN RDT (SD- Biosensor, India) was used for point-of-care detection of *P. falciparum* and/or other *Plasmodium* infections from a finger prick blood. At least 500 μl blood was taken into a microcuvette and post-centrifugation, the blood components were separated and stored at − 80 ℃. DNA was extracted from the red blood cells (RBCs). Laboratory-confirmed *Plasmodium* infections were those with parasites in peripheral blood as detected via RDT and/or polymerase chain reaction (PCR).

### Species-specific PCR detection of *Plasmodium* parasites

Genomic DNA was extracted from blood using the QIAamp® DNA Blood Mini Kit (QIAGEN, Germany) and eluted in 50 μl of distilled water. The species-specific nucleotide sequences of the 18S ribosomal RNA (18SrRNA) genes of *P. falciparum, P. vivax, P. malariae,* and *P. ovale* spp. were amplified in two successive rounds of PCR as described by Snounou [20]. For differentiation of two sub-species of *P. ovale* (*P. o. curtisi* and *P. o. wallikeri*), partial sequence of small subunit ribosomal (18 S rRNA) gene was amplified by using two-step PCR and confirmed by DNA sequencing. PCR products were visualized under UV light following electrophoresis on 1.2% (w/v) ethidium bromide-stained agarose gel and images captured using a gel documentation system.

Briefly, primary PCR was performed using PO1: CTACTTGACATTTCTACT TACA and PO2: CGTTCTTGATTAATGGAAGTAT and nested PCR was carried out using PO3: GCTGTAGCTAATACTTGCTTTA and PO4: TTCACCTCTGACATCTGAATC primers. The primary PCR was performed in a 25 μL reaction mixture that contained 0.2 U of Taq DNA polymerase (GCC Biotech, India), 0.2 mM each dNTP (HIMEDIA Laboratories, India), 0.4 mM each primer (GCC Biotech, India) and 2.0 mM MgCl_2_ (GCC Biotech, India). The reaction was allowed to proceed for 35 cycles after an initial denaturation at 94 ℃ for 1 min, annealing at 50 ℃ for 1 min and extension at 72 ℃ for 1 min; final extension was at 72 ℃ for 10 min in a Thermal Cycler (Agilent, USA). The nested PCR reaction condition was same as primary PCR except the annealing temperature which was 55 ℃.

PCR product of *P. ovale* spp.-positive samples were purified using QIA quick gel extraction kit (QIAGEN, Germany) following the protocol provided by the manufacturer and then subjected to DNA sequencing. 18 S rRNA gene was sequenced partially from both directions using the above-mentioned primers (PO3 and PO4) in a DNA Sequence Analyser (Applied Bio systems, Life Technology, USA). The consensus sequences were created after editing the DNA sequences using MultAlin (MultAlin Version 5.4.1, INRA, France). The sequences thus obtained were analysed by aligning with the available sequence at the National Centre for Biotechnology Information (NCBI) database for *P. o. wallikeri* (KF219558.1) and *P. o. curtisi* (KF696373.1).

## Results

Of the 3557 respondents from whom blood samples could be collected, 1,127 (31.7%) were male and 2,430 (68.3%) were female. The age of the study population ranged from 1 to 90 years. Overall, the median age was 24 years (IQR = 26 years) and the mean age was 23.12 years (± SD = 17.09). Amongst the individuals from whom blood samples were collected, 339 (9.5%) were pregnant and 724 (20.4%) were under five years old.

Of the 3557 blood samples, 170 (4.8%) were found to be RDT-positive for malaria parasites, whereas PCR successfully amplified *Plasmodium* DNA in 282 (7.9%) samples (Table 1). Amongst the RDT-negative samples, 50 (1.5%) were PCR-positive; amongst RDT-positive cases, 14 (8.2%) were PCR-negative. PCR identified substantially a higher number of *Plasmodium* carriages than RDT. Of the total PCR-positive samples, 168 (59.6%) were carrying mono-infection and 114 (40.4%) mixed infections. The majority of the cases with mono-infection were *P*. *vivax*, accounting for 40.8%. The *P. falciparum* mono-infection was present in 15.6% (44/282), *P. malariae* in 2.5% (7/282) and *P. ovale* spp. 0.7% (2/282) of the cases. Amongst the mixed infections 29.1% (82/282) were double infection, 10.3% (29/282) were triple infection, and 1.1% (3/282) quadruple infection. Out of 82 mixed infections *P. ovale* spp. was found in 27 (32.9%) of the cases and the majority (22 out of 27, 75.8%) were mixed with either *P. falciparum* and/or *P. vivax* (Table 2). Amongst the 29 *P. ovale* spp. PCR-positive individuals, 12 (41.4%) were clinically symptomatic, while 17 (58.6%) were clinically asymptomatic. Overall, the median age of the individuals positive for *P. ovale* spp. was 8 years (IQR = 22.5 years) and the majority were female (n = 17, 58.6%), two were pregnant/lactating. Asymptomatic malaria in this study was classified as a malaria infection (PCR positive) found in participants with axillary temperature < 37.5 °C at the time of the survey with absence of fever within the previous two days. Participants with asymptomatic malaria accounted for 55.67% (157/282) of the study population, and symptomatic 44.3% (125/282).

**Table 1:** Details of blood samples collection sites from six DAMaN-implemented districts of Odisha

District	Block	Blood specimen collected	RDT	PCR
Angul	Pallahara	298 (39.2%)	4 (1.3%)	4 (1.3%)
Angul	Chendipada	268 (34.3%)	3 (1.1%)	4 (1.5%)
Kalahandi	Biswanathpur	251 (38.0%)	23 (9.2%)	49 (19.5%)
Kalahandi	M. Rampur	181 (34.9%)	13 (7.2%)	10 (5.5%)
Kandhamal	G.udayagiri	320 (49.9%)	0 (0.0%)	0 (0%)
Kandhamal	Tikabali	296 (38.8%)	0 (0.0%)	0 (0%)
Keonjhar	Harichandanpur	402 (40.5%)	4 (1.0%)	4 (1%)
Keonjhar	Telkoi	208 (36.6%)	1 (0.5%)	0 (0%)
Rayagada	Chandrapur	331 (43.2%)	81 (24.5%)	102 (30.8%)
Rayagada	Kashipur	339 (52.5%)	15 (4.4%)	9 (2.7%)
Sundargarh	Lahunipara	292 (51.7%)	24 (8.2%)	100 (34.2%)
Sundargarh	Gurundia	371 (45.5%)	2 (0.5%)	0 (0%)
Total		3557	170	282

The results of two different diagnostic tests (RDT and PCR) are mentioned

**Table 2:** Different malaria species infection dynamics as mono or mixed infection in six districts of Odisha

District		Angul		Kalahandi		Kandhamal		Keonjhar		Rayagada		Sundargarh		Total
Block		Pallahara	Chendipada	Biswanathpur	M. Rampur	G.udayagiri	Tikabali	Harichandanpur	Telkoi	Chandrapur	Kashipur	Lahunipara	Gurundia	
Mono infection														
Pf		4	1	6	2	0	0	2	0	28	0	1	0	44
Pv		0	1	16	5	0	0	0	0	25	3	65	0	115
Pm		0	0	1	0	0	0	0	0	6	0	0	0	7
Po		0	0	1	0	0	0	0	0	1	0	0	0	2
Total mono infection		4	2	24	7	0	0	2	0	60	3	66	0	168
Total mixed infection (Pf, Pv/ Pf, Po/ Pv, Pm/ Pf, Pv, Pm / Pv, Pm, Po/ Pf, Pv, Po / Pf, Pv, Pm, Po)		0	2	25	3	0	0	2	0	42	6	34	0	114
*P ovale* spp found in mixed infection		0	1	12	0	0	0	0	0	8	0	6	0	27
*P malariae* found in mixed infection		0	1	4	0	0	0	0	0	9	0	10	0	24

The presence of *P. ovale* spp. malaria was detected in five community health centres (CHCs) belonging to four districts and three geophysical regions. Prevalence estimates ranged from a maximum of 12.9% (43 out of 331) in Chandrapur CHC of Raygada district to a minimum of 0.74% (2 out of 268) in Chhendipada CHC of Anugul district. Region-wise distribution indicated high prevalence of *P. ovale* spp. in Eastern ghat (Raygada and Kalahandi), Central table land (Anugul) and Northern plateau (Sundargarh) regions. However, *P. malariae* has been detected in three districts belonging to Eastern ghat (Raygada and Kalahandi) and Northern plateau (Sundargarh) regions.

**Fig. 2:**
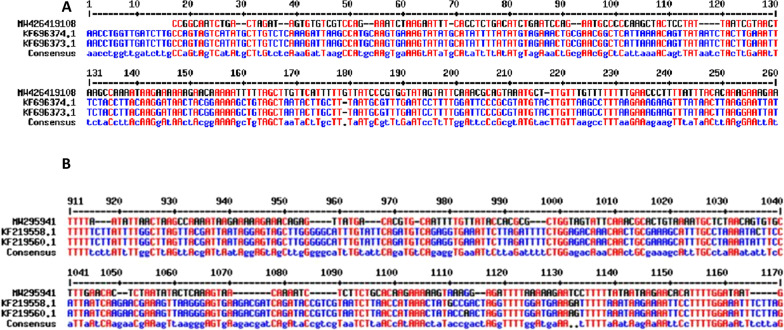
Nucleotide sequence of PCR amplified fragment of **A** *Plasmodium ovale curtisi* (MW426419108) and **B** *Plasmodium ovale wallikeri (MW295941)* 18 s r RNA gene, showing alignment with National Centre for Biotechnology Information (NCBI) database sequence for *P. ovale curtisi* (KF696374.1 and KF696373.1) and *P. ovale wallikeri* (KF219558.1 and KF219560.1) using multalin

Of the 29 samples that were PCR-positive for *P. ovale* spp*.,* 5 (17.2%) belong to *P.* *o. wallikeri*, 11 (37.9%) *P.* *o. curtisi* and 13 (44.8%) to both *P. o. wallikeri* and *P. o. curtisi*. DNA sequencing was carried out using PCR product of seven *P. ovale* spp.-positive samples, which were of sufficient quality and quantity for the detection of distinct *Plasmodium* spp. 18S haplotypes in an ABI-3730 XL DNA Analyser (Applied Biosystems). Obtained DNA sequences after editing were analysed by aligning with the sequence available at the NCBI Gene Bank for *P. o. curtisi* and *P. o. wallikeri*. The sequence data have been submitted to the NCBI data base and the GenBank Accession No of *P. o. curtisi* is MW426407/MW 426,410 /MW426419/MW426434/W426443 and *P. o. wallikeri* is MW295851/MW295941 (Fig. 2).

## Discussion

This study has demonstrated, the presence of *P. ovale* spp*.* in different geophysical regions of Odisha, an eastern Indian state. In contrast, the previous studies in India reported presence of *P. ovale* spp*,* in few individuals or restricted to certain foci. The majority of *P. ovale* spp. infections observed were mixed with either *P. falciparum* and/or *P. vivax*. Molecular typing of *P. ovale* spp. indicated sympatric distribution of the sibling species of *P.* *o. wallikeri* and *P.* *o. curtisi.* In addition, the study recorded prevalence of *P. malariae*, another rare malaria parasite in 10.3% of positive samples (irrespective of their status of single or mixed infections). PCR assay has been used to obtain a high sensitivity and specificity in detection of both mixed-species and non-falciparum infections.

Although generally considered benign, *P*. *ovale* spp. and *P.* *malariae* have the potential to cause significant morbidity. Recently reports of *P. ovale* spp. infection with acute respiratory distress syndrome (ARDS) [8], acute renal failure [21] and splenic complications [22] have drawn the attention of public health researchers. *Plasmodium ovale* spp. has limited distribution, with endemic transmission traditionally limited to areas of tropical Africa, New Guinea, the eastern parts of Indonesia and the Philippines. Infections with *P. ovale* spp. however, have also been reported in the Middle East and different parts of Southeast Asia [23]. With respect to the Indian sub-continent, cases of *P. ovale* spp. has been reported from Bastar Division of Madhya Pradesh in 2017 [13] and three cases in Koraput District of Odisha in 1989 [24]. However, detection of *P. ovale* spp. infections during the current survey, using PCR in a wide geographical area (covering north to south) of a state that has been experiencing substantial reduction of *P*. *falciparum* infection lately, might be one of the risk factor in persistence of malaria in the future. Furthermore, prevalence of *P.* *ovale* spp. infections almost exclusively as mixed species infections with *P. falciparum* and/or *P. vivax* rather than mono-infections, illustrates that the transmission of *P. ovale* spp. followed those of *P. falciparum* and/or *P. vivax*. Identification of the sibling species *P.* *o. curtisi* and *P.* *o. wallikeri* of *P. ovale* alone and in combination during the present study can conclude that there is no general physical or temporal barrier preventing recombination of these related parasite species, but rather that *P. o. curtisi* and *P. o. wallikeri* maintain their distinct genetic identity through yet unknown biological mechanisms [25]. Moreover, *P. malariae* infections have been observed in all major malaria-endemic regions of the world [26] including India. Earlier studies in Odisha have reported similar, relatively higher prevalence of *P. malariae* (9.1% mono-infection and 10.9% mixed with either *P. falciparum* and/or *P. vivax*) [26] in comparison to that of *P. ovale* spp. that was currently observed in the DAMaN assessment survey.

Diagnosis of *P. ovale* spp. and *P. malariae* can be done by microscopy but is often confused with *P. vivax.* RDTs are even less sensitive for detection of non-falciparum malaria (NFM) as compared to microscopy. However, PCR, being most sensitive for such purpose, has been recognized as a valuable tool for confirming or diagnosing NFM, especially in less-endemic regions [27]. According to current WHO guidelines, primaquine treatment is recommended to prevent relapses of *P*. *ovale* spp. infections [28]. The first-line anti-malarial treatment used at the study site was chloroquine for vivax malaria and artemisinin-based combination therapy (ACT) for falciparum malaria as per NVBDCP guidelines. ACT was readily available in the villages where the survey was conducted, as these pockets were mostly ravaged by falciparum malaria. It is well known that ACT, meant for targeting *P*. *falciparum* in national programmes, is also highly efficacious against asexual stages of both *P*. *malariae* and *P*. *ovale* spp. However, to prevent relapses of malaria from these two rarer/neglected species, radical treatment with primaquine is necessary, or else they will remain in circulation and continue to fuel the malaria problem. This can only be undertaken when the programme can detect the rarer/neglected species routinely, the modalities of which may be studied through an implementation research framework. However, currently there is no provision for this, and rightly so, because the rarer/neglected species are yet to caste a considerable burden on malaria scenario in the country. The current study demonstrates that as India gears up to eliminate malaria by 2030, the burden of malaria due to rarer/neglected species can assume substantial proportions in the face of dwindling rates of falciparum and vivax malaria. This study informs the national programme of what might be a future challenge, so that pre-emptive measures can be instituted.

The findings highlight some of the key challenges that need to be addressed if malaria elimination is to be achieved in the Indian sub-continent. The observed high and wide prevalence of *P*. *ovale* spp and *P.* *malariae* in a geographical area where the prevalence of *P*. *falciparum* has declined may support previously raised concerns that strategies designed for reducing transmission of *P*. *falciparum* may be less effective in reducing transmission of the non-falciparum species of *Plasmodium* [6]. Further studies will be crucial for confirming if control and elimination strategies targeting *P. falciparum* and *P. vivax* are as effective with *P. ovale* spp. and *P. malariae* or if additional strategies will be necessary to eliminate these broadly distributed and chronically under-detected rarer/neglected species of malaria. The current malaria programme of India may have to be redesigned with newer diagnostic and surveillance tools and treatment protocols to address rarer/neglected malaria species, especially when *P. falciparum* and *P. vivax* spp. are in rapid decline. Microscopy and currently available RDTs have their merits and demerits in diagnosis of *P. ovale* spp. and *P. malariae*. However, molecular-based diagnostic approach is a highly sensitive and specific method to determine the molecular signature of all human *Plasmodium* spp. During the COVID-19 pandemic all district headquarter hospitals (DHHs) have established molecular diagnostic facility and deployed trained human resources for diagnosis and reporting of SARS-CoV-2 infection in resource-prone states such as Odisha. These laboratories can be utilized as a central facility in future for diagnosis and confirmation of malaria species, particularly rare species, in those high-burden malaria districts. In the absence of proper diagnosis of malaria species, the ambitious goal of malaria elimination may otherwise be derailed by a surge in these rarer species.

## Conclusions

The study presents to the malaria control community the wide distribution of *P. ovale* spp. and *P. malariae* in different geophysical regions of the state of Odisha. This finding may spur incorporation of appropriate surveillance strategies by the national malaria programme to detect such rarer/neglected species in the community. That may further lead to institution of newer treatment modalities to combat these parasites within the framework of the programme, so that comprehensive malaria elimination can be achieved in India.

## Data Availability

All data generated or analysed during this study are included in this published article.
